# Supplementary material for: Taxifolin as a Metallo-β-Lactamase Inhibitor in Combination with Augmentin against Verona Imipenemase 2 Expressing Pseudomonas aeruginosa
Source: Microorganisms. 2023 Oct 28;11(11):2653. doi: 10.3390/microorganisms11112653 (PMC10673258; doi:10.3390/microorganisms11112653)

SUPPLEMENTARY INFORMATION

Taxifolin as a metallo-β-lactamase inhibitor in combination with Augmentin against VIM-2-expressing *Pseudomonas aeruginosa*.

Bogdan M. Benin[1], Trae Hillyer[1], Aylin S. Crugnale[1], Andrew Fulk[1], Caitlyn A Thomas[2], Matthew A. Smith[1,3], Michael W. Crowder[2], Woo Shik Shin[1*]

[1]Department of Pharmaceutical Sciences, Northeast Ohio Medical University, Rootstown, OH 44272

[2]Department of Chemistry and Biochemistry, Miami University, Oxford, OH 45056

[3]Akron Children's Hospital, Rebecca D. Considine Research Institute, Akron, OH 44302, United States

[*]Corresponding Author: Woo Shik Shin

E-mail: wshin@neomed.edu

Telephone: (330) 325-6449

Fax: (330) 325-5936

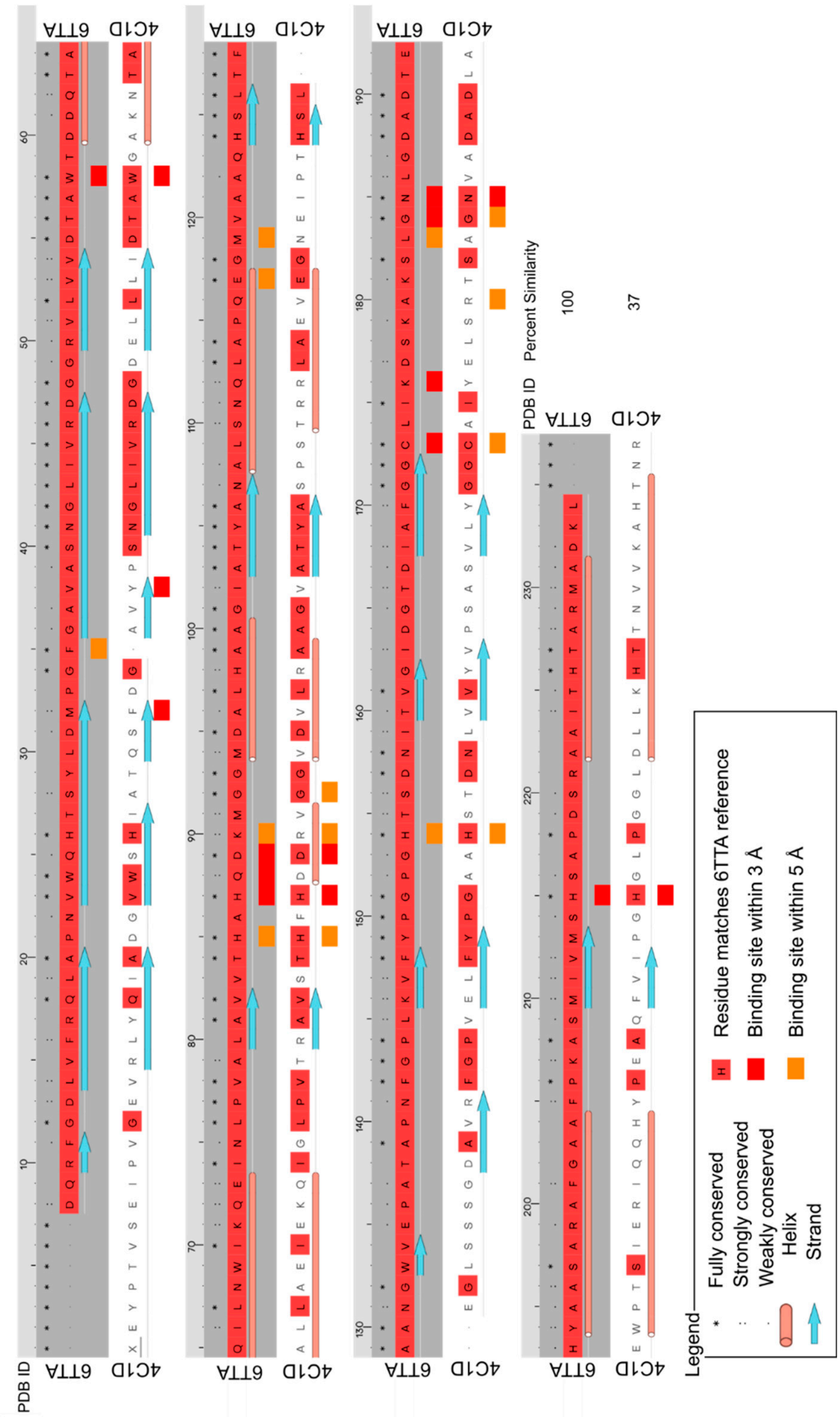

Supplementary Figure S1. Multiple sequence viewer comparison of NDM-1 (6TTA) and VIM-2 (4C1D) amino acid sequences. Comparing both sequences demonstrates that the active site of these enzymes is highly conserved as indicated by the colors and symbols in the legend. Significant differences between these enzymes are observed at chains more than 5 Å away from the binding site.

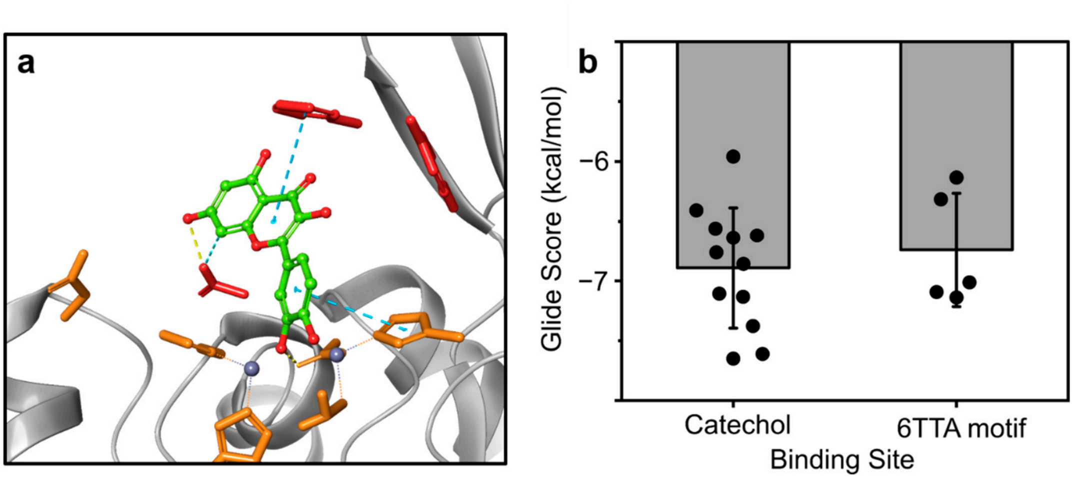

Supplementary Figure S2. Catechol binding pose is predicted to potentially occur in quercetin/VIM-2 inhibition. (a) Predicted docking model of quercetin with VIM-2 (PDB:4C1D). (b) Comparison of the two primary binding modes found for quercetin; the "6TTA motif" refers to the binding of the keto-enol type moiety to the Zn ions in the active site.

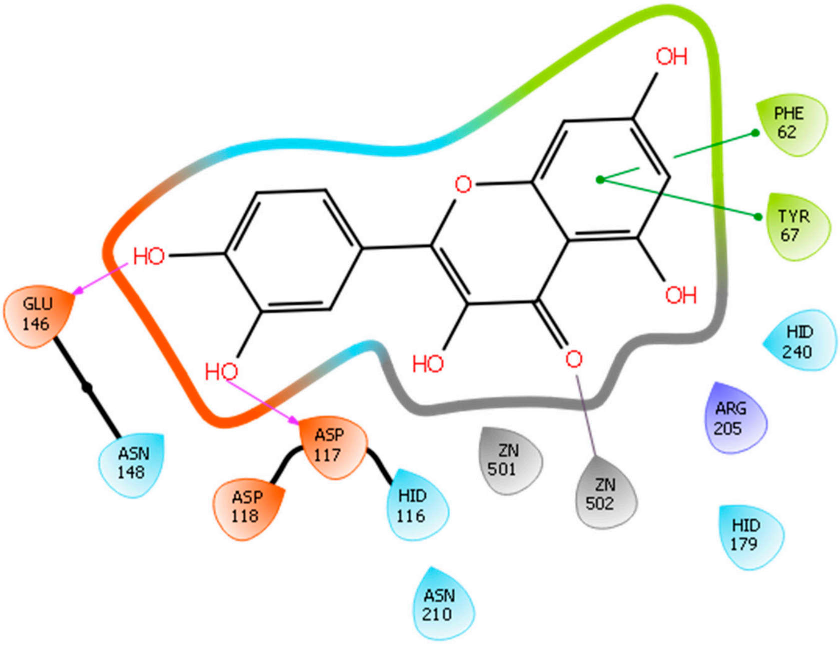

Supplementary Figure S3. Ligand interaction diagram for quercetin bound to VIM-2. Orange indicates negatively charged residues, light blue indicates polar residues, grey indicates the metal Zn ions, green indicates hydrophobic residues, and the cyan color indicates negatively charged residues. Purple arrows denote hydrogen bonds, while green lines show aromatic interactions.

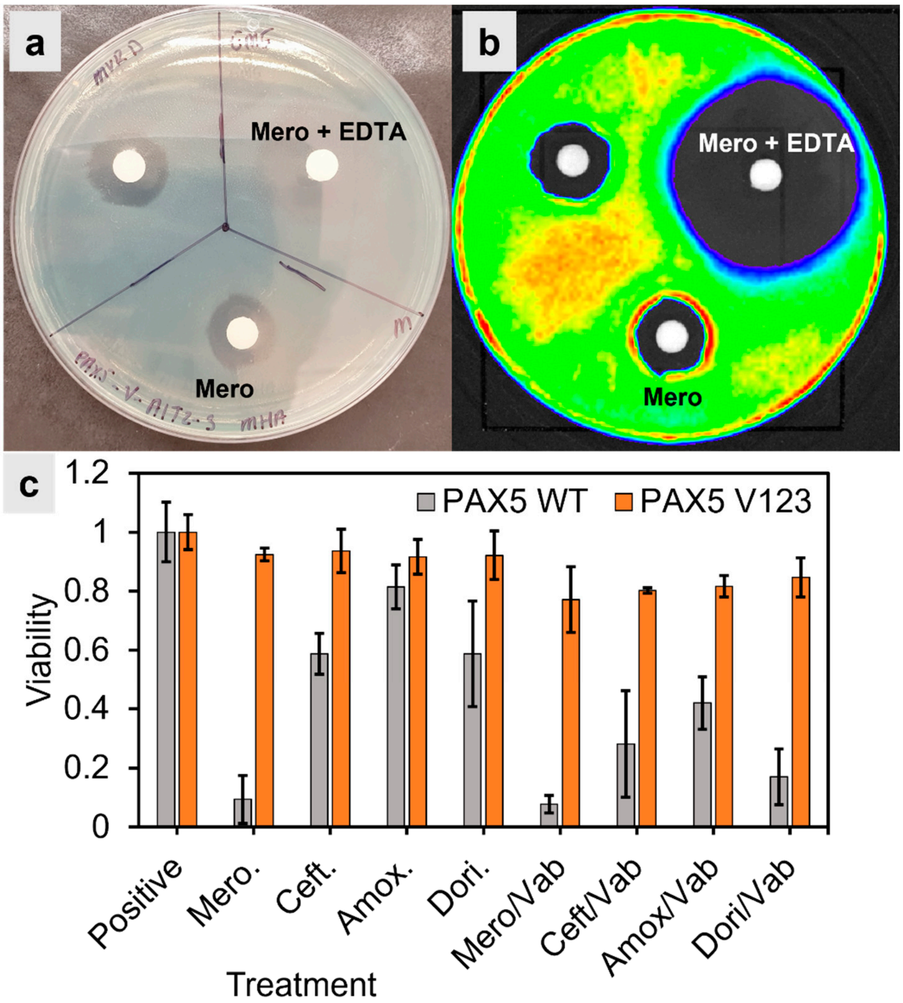

Supplementary Figure S4. Transformation and single dose testing of VIM-2 expressing BLPA. (a) Photograph of disc-diffusion assay comparing inhibition zones of meropenem and meropenem with EDTA against $BLPA_{VIM-2}$ on Müller-Hinton agar plates. (b) Luminescent image of the same plate demonstrating that bacteria retain bioluminescence. (c) Single dose assay comparing the VIM-2 expressing BLPA with the wild type against several carbapenems as well as carbapenems combined with vaborbactam.

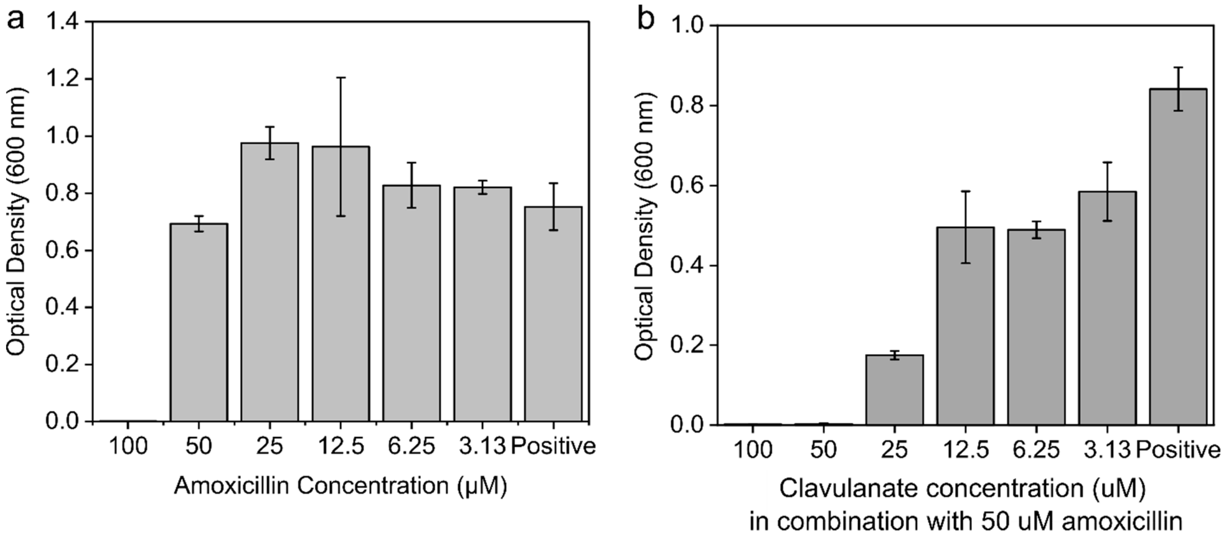

Supplementary Figure S5. MIC vs. BLPA$_{VIM-2}$ of amoxicillin (a) and clavulanate (b).

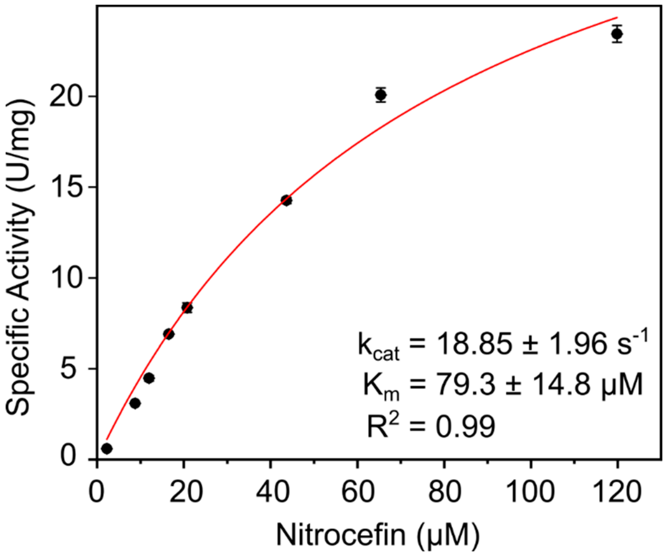

Supplementary Figure S6. VIM-2 enzyme kinetics. Nitrocefin was utilized as the substrate as its hydrolysis product can be measured at 486 nm.

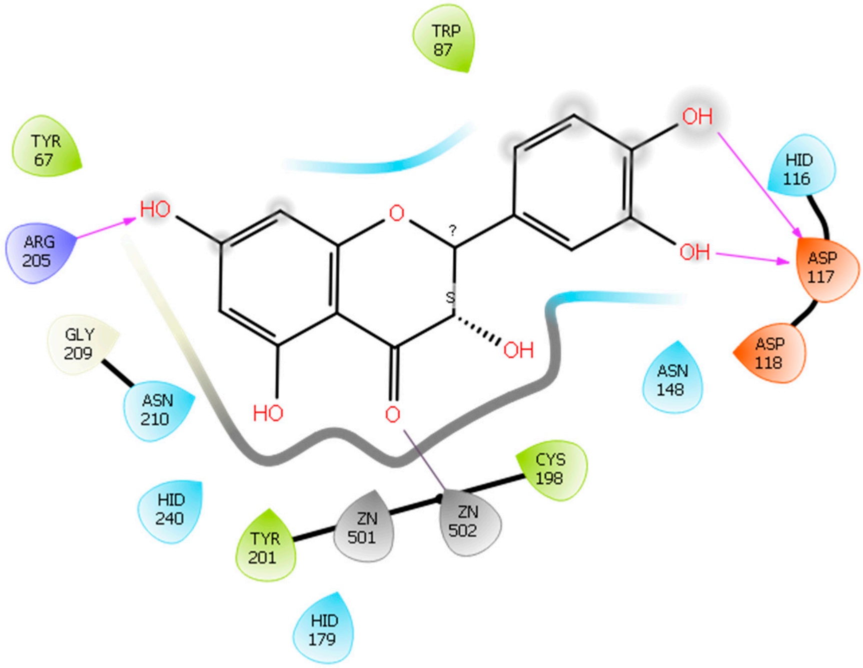

Supplementary Figure S7. Ligand interaction diagram for 5 (taxifolin) bound to VIM-2. Orange indicates negatively charged residues, light blue indicates polar residues, grey indicates the metal Zn ions, green indicates hydrophobic residues, and the cyan color indicates negatively charged residues. Purple arrows denote hydrogen bonds, while green lines show aromatic interactions.

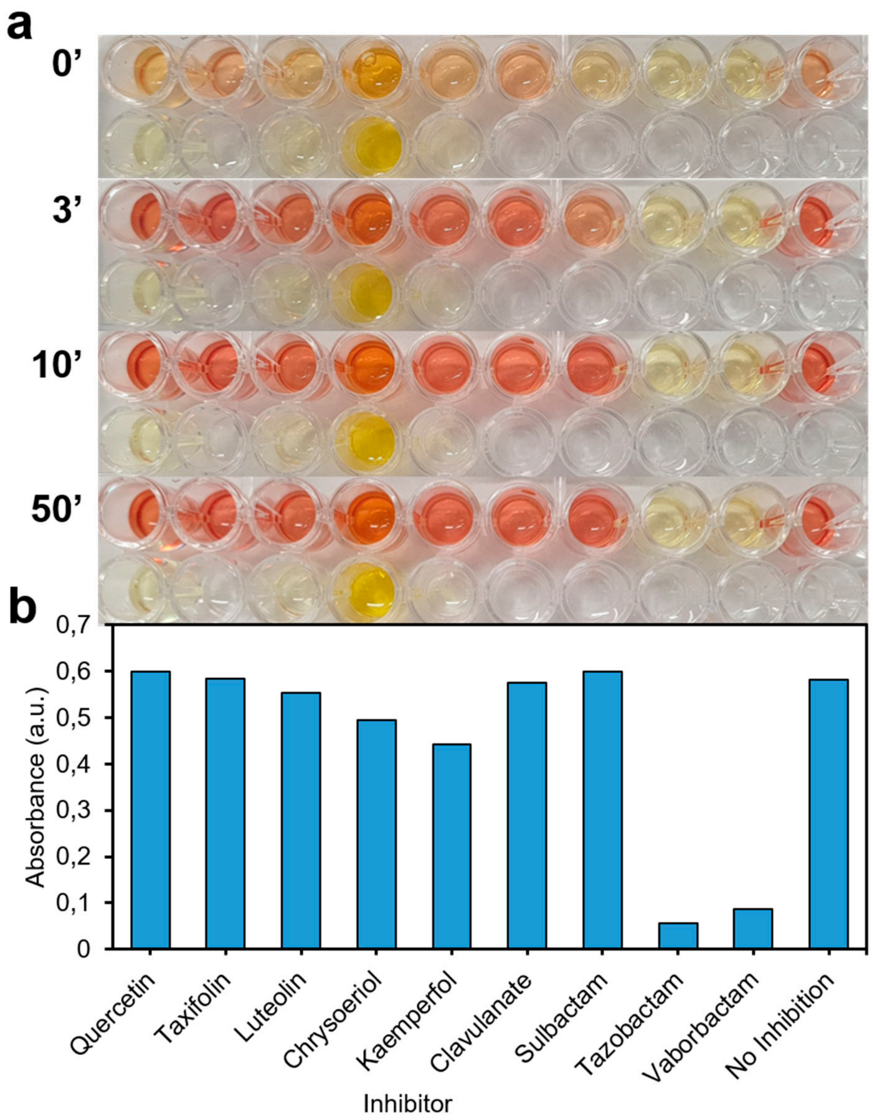

Supplementary Figure S8. Single dose inhibition of quercetin analogues against serine-β-lactamase, TEM-1. (a) Photographs of well plates at various time points containing inhibitors, TEM-1, and nitrocefin. The columns correspond to the treatments listed in the x-axis in subfigure b. Bottom rows were used as blank controls to take into account the colored appearance of flavinol compounds. (b) Absorbance readings at 486 nm after 50 minutes.

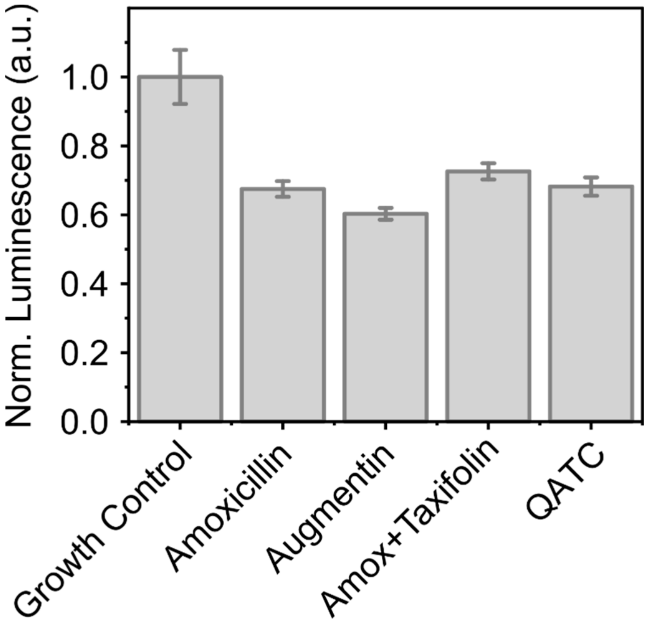

Supplementary Figure S9. Single dose inhibition of taxifolin against SBL-producing PAX5-WT. Concentration of amoxicillin was 25 µM for all samples. The molar ratio of the augmentin was 2:1 (amoxicillin:clavulanate); the molar ratio of the amoxicillin:taxifolin was 1:1; the molar ratio of the QATC was 2:1:2 (amoxicillin:clavulanate:taxifolin).

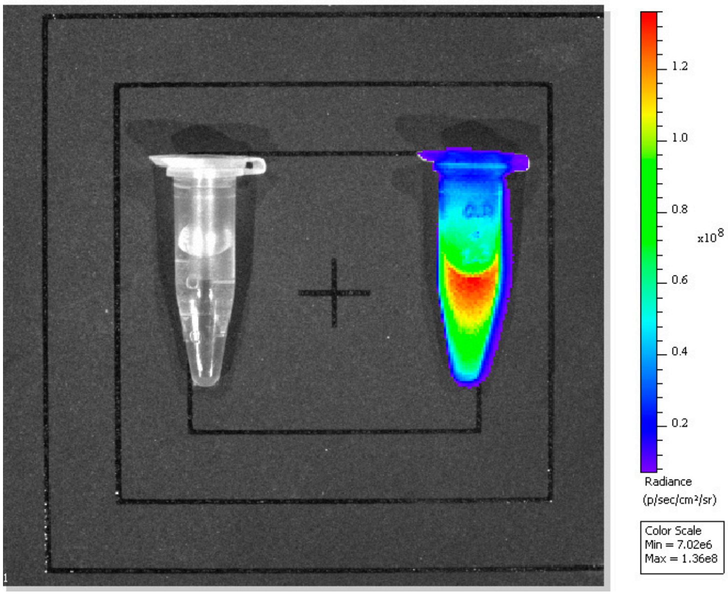

Supplementary Figure S10. IVIS image of PBS (left) and PAX5$_{VIM-2}$ (right) used to inoculate mice.

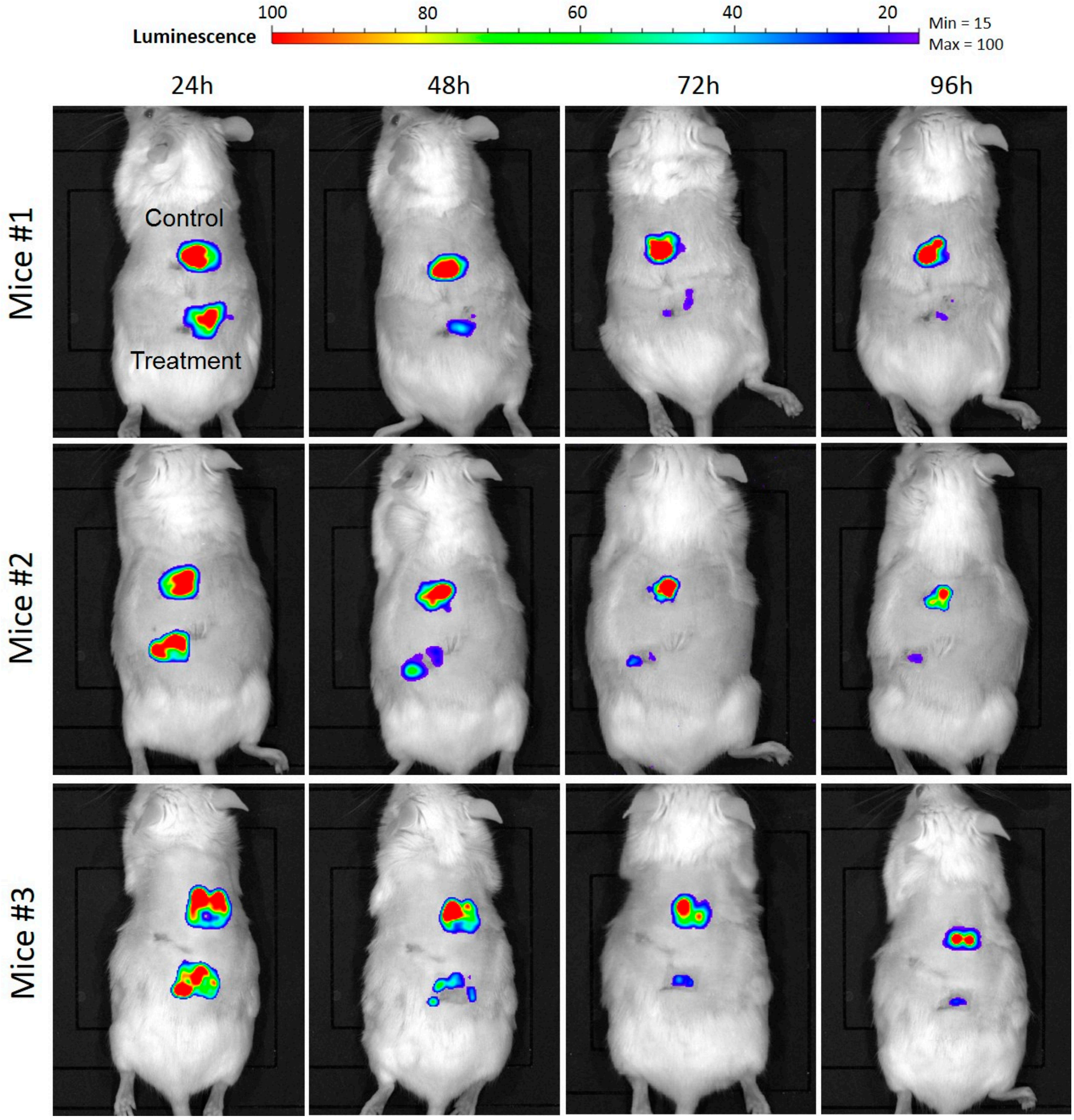

Supplementary Figure S11. *In-vivo* treatment of murine wound models infected with PAX5$_{VIM-2}$. IVIS imaging of local skin wound infection by bioluminescent P. aeruginosa (Xen05, VIM-2(+)) treated with either vehicle control, or Triple combination treatment (Augmentin + taxifolin) over four days.

Supplementary Note S1:

Each 1cm^2 square contains numerous needle scratches created with a 25 gauge needle. These are made gradually by repeated scratching in horizontal and vertical directions, until the wound within the square takes on an evenly red and inflammed appearance (the image on the right demonstrates the correct appearance of needle scratches). Caution should be given to the pressure applied during this process in order to avoid deep incisions into the subcutanouse layer, which can happen easily. If active bleeding occurs, or if it is clear that a deep incision into the subcutanous tissue has been created, it may be best to avoid infection on that wound and allow the animal to recover following the use of an appropriate antibiotic spray. One of our rodents with an accidental subcutaneous incision died shortly after the bacterium had been applied. The autopsy showed a deep skin infection which directly spread through the muscles to the lungs. Together with the ischemic tissue of the kidney, the rodent most likely developed a systemic spread of the wound infection with subsequent multiorgan failure and death within 2 days.

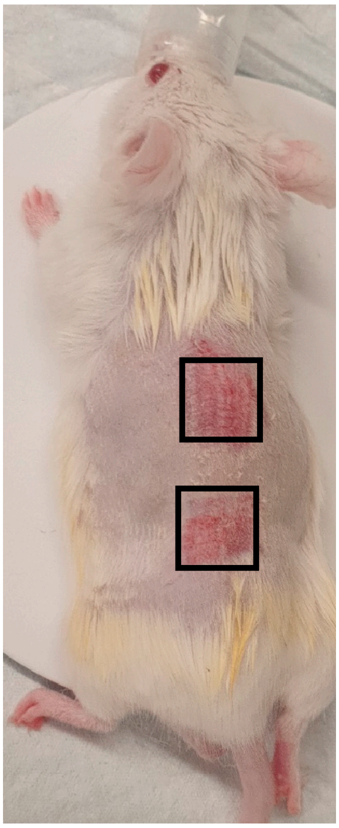

Supplement: Supplementary file 1 [file microorganisms-11-02653-s001.zip › microorganisms-2640975-supplementary.pdf]
